# Genomic detection of highly pathogenic avian influenza H5N1 in Antarctic seabirds reveals connectivity with South American viral lineages

**DOI:** 10.1007/s42770-026-02076-7

**Published:** 2026-09-14

**Authors:** Francine C. B. Timm, Raíssa Gasparetto, Miguel L. Côrrea, Alanis S. Melgarejo, Bruna S. Paredes-Galarza, Nicole V. Stone, Eduardo J. Pizarro, Gaspar Mejías, Carolina P. Lucca, Isabella Grazini-Capelin, Fernanda M. Pereira, Luis G. Santos, Thales Bermann, Bruno A. Prandi, Lina M. Violet-Lozano, Martha T. Oliveira, Lucas Krüger, Ana C. Franco

**Affiliations:** 1 Laboratory of Virology, Department of Microbiology, Immunology and Parasitology, Institute of Basic Health Sciences, Federal University of Rio Grande do Sul, Porto Alegre, Rio Grande do Sul Brazil; 2 Millennium Institute Center for Genome Regulation (CGR), Santiago, Chile; 3 Millennium Institute Biodiversity of Antarctic and Subantarctic Ecosystems (BASE), Santiago, Chile; 4 Chilean Antarctic Institute, Santiago, Chile

**Keywords:** avian influenza virus, Antarctic seabirds, H5N1, wildlife surveillance, Antarctic

## Abstract

Emerging avian viruses increasingly threaten Antarctic wildlife, raising concerns about ecosystem health and biodiversity. In this study, we conducted a comprehensive investigation of avian influenza virus (influenza A virus, IAV) in both resident and migratory birds inhabiting the South Shetland Islands, Antarctica. During the 2024–2025 austral summer, 278 samples were collected and screened using real-time RT-PCR targeting the IAV M gene. IAV RNA was detected in 30 samples, and eight of these were found to be positive for H5. Complete genome sequencing was performed on samples from a gentoo penguin (*Pygoscelis papua*) and a southern giant petrel (*Macronectes giganteus*), revealing the presence of highly pathogenic avian influenza virus H5N1, clade 2.3.4.4b. Phylogenetic analysis demonstrated that these viral genomes closely cluster with contemporary South American strains, indicating a direct connectivity between Antarctic seabirds and the broader H5N1 transmission network. Our findings highlight the heightened vulnerability of Antarctic ecosystems to emerging infectious diseases and emphasize the critical need for sustained genomic surveillance. These efforts are essential to monitor wildlife health, inform conservation strategies, and implement effective biosecurity measures to safeguard Antarctic biodiversity.

**Supplementary Information:**

The online version contains supplementary material available at https://doi.org/10.1007/s42770-026-02076-7.

## Introduction

Around 100 million birds breed in the Antarctic Peninsula during the austral summer, including endemic and migratory species [1]. Although Antarctica is geographically remote and characterized by extreme environmental conditions, it is increasingly recognized as a relevant system for studying wildlife health and emerging infectious diseases, particularly in the context of accelerating climate change [2]. Recently, the global spread of highly pathogenic avian influenza viruses (HPAIVs) has intensified concerns regarding viral introduction into Antarctic ecosystems [3]. Migratory birds traveling across intercontinental flyways connect polar, temperate, and tropical regions and play a key role in the long-distance dissemination of avian influenza viruses, including to Antarctica [4–7], where animal populations may be particularly susceptible due to limited historical contact with such viruses and, consequently, a lack of pre-existing immunity [8–10].

Before the emergence and recent spread of highly pathogenic avian influenza in South America, surveillance studies conducted in the Antarctic Peninsula reported limited or no detection of respiratory viruses in birds and marine mammals [11]. Nonetheless, circulation of influenza A virus (IAV) had already been documented, including the identification of subtypes such as H11N2 in penguin fecal samples [11]. Besides, subsequent molecular surveillance confirmed additional IAV subtypes, including H1N1 and H3N8, on King George Island in 2023, revealing genetic heterogeneity of viruses circulating in Antarctic wildlife [12]. Finally, after years of concern regarding potential viral introduction, the first detection of HPAIV in Antarctica occurred in 2023, when H5N1 was identified in a brown skua (*Stercorarius antarcticus*) on Bird Island, South Georgia [3]. This event was followed by rapid southward spread, with HPAIV H5 reaching mainland Antarctica by December 2023 and causing mortality events among seabirds and marine mammals [13]. Genomic analyses of H5N1 clade 2.3.4.4b isolated from brown skuas in 2024 suggested introduction via South America [14].

Influenza A viruses (IAV; genus *Alphainfluenzavirus*, family *Orthomyxoviridae*) comprise a genetically diverse group of negative-sense, single-stranded RNA viruses that infect a broad range of avian and mammalian hosts [15]. To date, 19 hemagglutinin and 11 neuraminidase subtypes have been described, with extensive genomic reassortment contributing to viral diversity and adaptability [16]. Among these, subtypes H1–H16 and N1–N9 circulate predominantly in wild and domestic birds, with aquatic birds—particularly Anseriformes and Charadriiformes—serving as the primary natural reservoirs [17, 18]. In wild birds, avian influenza viruses may cause infections ranging from asymptomatic to severe disease, and viruses are classified as low pathogenicity or highly pathogenic based on their virulence in poultry [19–20]. While low-pathogenic viruses typically circulate at low prevalence in Antarctic avifauna, some H5 and H7 lineages may evolve into highly pathogenic forms capable of causing severe disease and mortality [3, 21].

Although Antarctic ecosystems were long considered insulated from large-scale epizootics, seabirds such as penguins, skuas, sheathbills, and migratory petrels interact with global avian flyways and may introduce and disseminate avian influenza virus lineages [1, 22, 23]. Previous detections of low-pathogenic avian influenza virus (LPAIV) subtypes at low prevalence (< 5%) suggest established but limited viral circulation in Antarctic bird populations [1, 3]. In contrast, the recent emergence and spread of HPAIV H5N1 in the region demonstrate that Antarctic wildlife is increasingly exposed to globally circulating viruses, with documented impacts on seabirds and marine mammals [3, 13].

These findings, together with the ongoing global emergence of novel avian influenza virus variants, highlight the importance of continued surveillance in Antarctic bird populations to detect viral incursions and assess risks to wildlife and interconnected ecosystems. Because influenza A viruses pose a significant public health threat due to their pandemic potential, integrated surveillance approaches that include wildlife reservoirs are essential for early detection and risk mitigation [24]. Therefore, the aim of this study was to investigate the occurrence and molecular characterization of influenza A virus in Antarctic birds sampled during the austral summer of 2024–2025 in the South Shetland Islands.

## Materials and methods

### Sample collection and study area

**Fig. 1:**
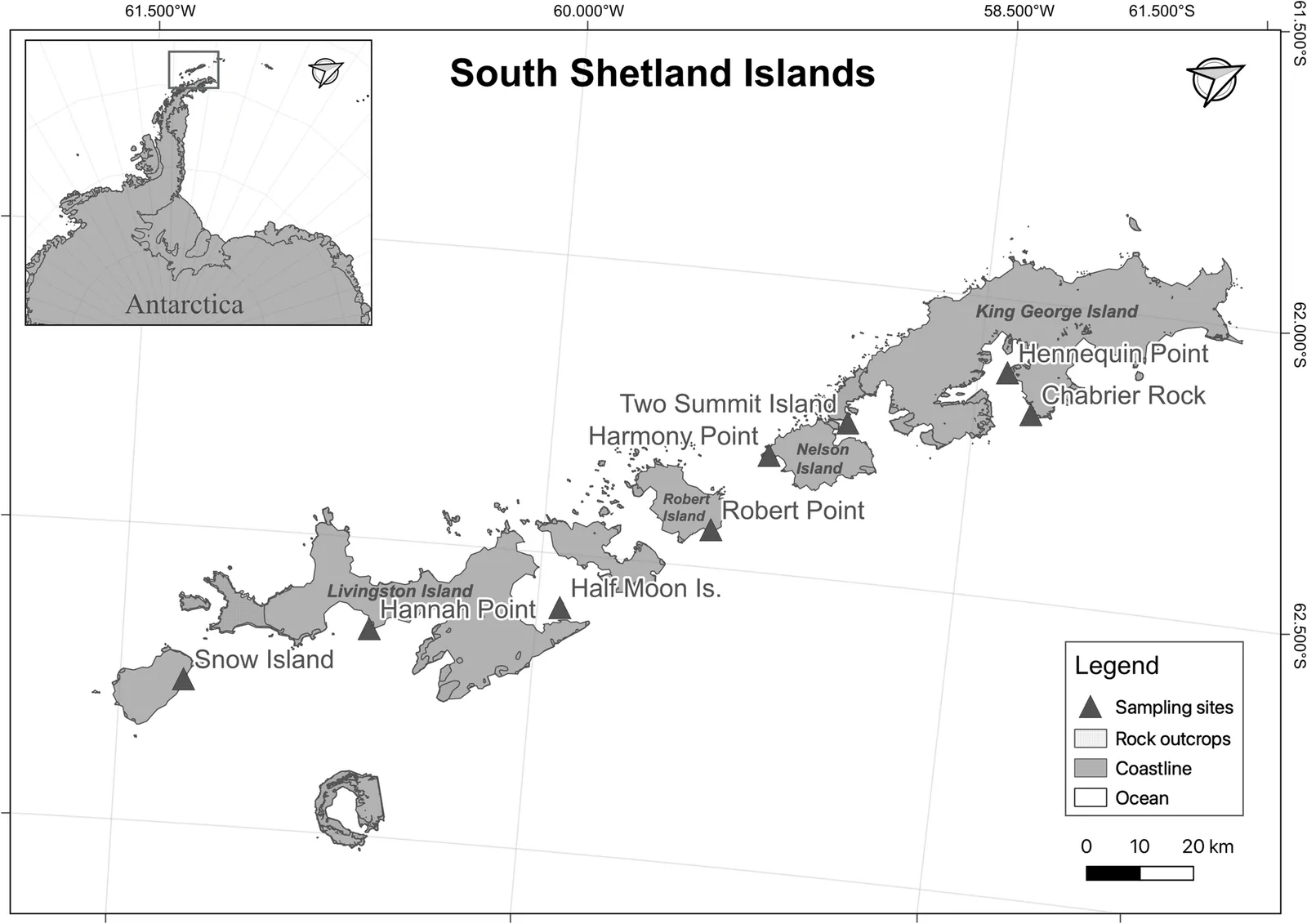
Study area in the South Shetland Islands, Antarctica, showing the sampling sites at seabird breeding colonies across King George, Half Moon, Robert, Livingston, Snow, and Nelson Islands. Pie charts indicate the proportion of IAV-positive (red) and IAV-negative (grey) samples. Base map source: SCAR Antarctic Digital Database (ADD v7.11) compiled by the British Antarctic Survey, obtained via the Quantarctica data package (Norwegian Polar Institute) and projected in WGS 84 / Antarctic Polar Stereographic (EPSG: 3031)

During the 43rd Antarctic Operation (XLIII OPERANTAR), 278 samples were collected from Antarctic avifauna across the South Shetland Islands, including King George, Half Moon, Robert, Livingston, Snow, and Nelson Islands (Fig. 1; Table 1). Samples comprised 166 cloacal excreta samples and swabs from 56 individuals (56 cloacal swabs and 56 orotracheal swabs). Sampling was conducted at breeding colonies of southern giant petrels (*Macronectes giganteus*), gentoo penguins (*Pygoscelis papua*), and chinstrap penguins (*Pygoscelis antarcticus*) and polar skua (*Stercorarius maccormicki*).

**Table 1:** Sampling sites and geographic coordinates of seabird colonies sampled during the 2024–2025 austral summer in the South Shetland Islands, Antarctica

Collection site^a^	Geographic coordinates^b^
Chabrier Rock (King George Is.)	62°11′12.96″S, 58°17′49.99″W
Half Moon Is.	62°35′23.25″S, 59°55′4.92″W
Hannah Pt. (Livingston Is.)	62°39′5.17″S, 60°36′13.90″W
Harmony Pt. (Nelson Is.)	62°18′8.49″S, 59°12′58.45″W
Hennequin Pt. (King George Is.)	62°7′18.68″S, 58°23′51.47″W
Robert Pt. (Robert Is.)	62°26′10.23″S, 59°23′55.33″W
Snow Is.	62°45′26.83″S, 61°16′4.01″W
Two Summit Is.	62°14′5.91″S, 58°56′48.42″W

^a^ *Is*. Island, *Pt*. Point, *Pen*. Peninsula. ^b^ Coordinates are provided in degrees, minutes, and seconds (*DMS*)

Virological analyses were performed at the Virology Laboratory, Institute of Basic Health Sciences, Federal University of Rio Grande do Sul, Brazil. All sampling procedures were conducted under institutional and national permits and complied with international biosafety and wildlife handling regulations, including the Scientific Committee on Antarctic Research (SCAR) guidelines for handling highly pathogenic avian influenza–positive samples in the Southern Ocean [25].

### RNA extraction and influenza A virus detection

Total RNA was extracted using the MagMAX™ CORE kit (Applied Biosystems), adapted for fecal matrices. Approximately 0.3 g of sample material was suspended in 1 mL of phosphate-buffered saline (PBS), vortexed, and clarified by centrifugation at 15,000 × g for 1 min. A 200-µL aliquot of supernatant was subjected to automated extraction on a KingFisher™ Flex platform, and RNA was eluted in 50 µL and stored at − 80 °C until analysis.

Complementary DNA (cDNA) was synthesized using a High-Capacity Reverse Transcription Kit following the manufacturer’s instructions. Real-time PCR assays were performed using TaqMan™ Fast Advanced Master Mix on a QuantStudio™ 1 Real-Time PCR System. Cycling conditions consisted of 50 °C for 2 min, 95 °C for 20 s, followed by 40 cycles of 95 °C for 1 s and 60 °C for 20 s, with fluorescence acquisition during annealing/extension.

Influenza A virus screening targeted the matrix (M) gene (101 bp) by real-time RT-PCR, following Spackman et al. [26]. Samples positive for the M gene were subsequently tested for the H5 hemagglutinin gene (131 bp; primers listed in Table 2) [27]. Cycle threshold (Ct) values < 30 were interpreted as high viral load, Ct values of 30–36 as low positive, and Ct values > 36 as negative unless a reproducible sigmoidal amplification curve was observed. Analytical sensitivity was evaluated using a TOPO TA plasmid containing the IAV M gene, tested in a nine-point serial dilution (10⁹–10¹ copies µL⁻¹).

**Table 2:** Primer and probe sequences used for RT-qPCR detection of AIV matrix and H5 genes

Primers	Sequences^c^	Amplicon size (bp)
M + 25	5’ - AGA TGA GTC TTC TAA CCG AGG TCG – 3’	101
M – 124	5’ - TGC AAA AAC ATC TTC AAG TCT CTG – 3’	
M + 64	FAM - TCA GGC CCC CTC AAA GCC GA-BHQ1	
H5v2 F	5’ – CGATCTAGAYGGGGTGAARCCTC – 3’	131
H5v2 R	5’ – CCTTCTCCACTATGTANGACCATTC – 3’	
H5v2 Pr	FAM – AGCCAYCCAGCTACRCTACA – BHQ1	
H5- 2010 F	5’ – CGATCTAAATGGAGTGAAGCCTC – 3’	
H5- 2010 R	5’ – CCTTCTCTACTATGTAAGACCATTC – 3’	
H5-2010 Pr	FAM – AGCCATCCCGCAACACTACA – BHQ1	

^c^ FAM, 6-*carboxyfluorescein*; BHQ1, *Black Hole Quencher 1*

### Subtyping and amplification of influenza A virus gene segments

All samples positive for IAV were subjected to PCR amplification of viral gene segments for sequencing. Primers targeting the hemagglutinin (HA) and neuraminidase (NA) genes, as well as subtype-specific primers for neuraminidase subtypes, were used (Table 3). PCR products were visualized by agarose gel electrophoresis and purified before sequencing.

**Table 3:** Primers used for amplification of all segments of avian influenza virus (AIV)

Primers	Sequences
HA F	5’ - TAT TCG TCT CAG GGA GCA AAA GCA GG*G G* – 3’
HA R	5’ - ATA TCG TCT CGT ATT AGT AGA AAC AAG G*GT GGT TT* – 3’
NA F^d^	5’ - TAT TGG TCT CAG GGA GCA AAA GCA GG*A GT* – 3’
NA R	5’ - ATA TGG TCT CGT ATT AGT AGA AAC AAG G*AG TTT TTT* – 3’
N3	5’ - TAT TCG TCT CAG GGA GCA AAA GCA GG*T GC* – 3’ 5’ - ATA TCG TCT CGT ATT AGT AGA AAC AAG G*TG C*TT TTT – 3’
N6	5’ - TAT TCG TCT CAG GGA GCA AAA GCA GG*G TG*A AAA TG – 3’ 5’ - ATA TCG TCT CGT ATT AGT AGA AAC AAG G*GT GGT TT* – 3’
N7	5’ - TAT TCG TCT CAG GGA GCA GCA AAA GCA GG*G TG*A TTG AGA ATG – 3’ 5’ - ATA TCG TCT CGT ATT AGT AGA AAC AAG G*GT GGT TT* – 3’
N9	5’ - TAT TCG TCT CAG GGA GCA AAA GCA GG*G TC* – 3’ 5’ - ATA TCG TCT CGT ATT AGT AGA AAC AAG G*GT CTT* – 3’

^d^Primer pairs used for specific amplification of *N1*, *N2*, *N4*, *N5*, and *N8* subtypes.

### Whole-genome sequencing of influenza A virus–positive samples

A subset of IAV–positive samples was selected for whole-genome sequencing. Library preparation, quality control, and sequencing were performed using the Ion Torrent platform. Raw sequencing reads were processed and assembled using the Iterative Refinement Meta-Assembler (IRMA) FLU pipeline developed by the Centers for Disease Control and Prevention, which performs reference-guided iterative assembly and consensus sequence generation for influenza virus genomes [28]. Consensus sequences for the HA and NA gene segments were obtained for downstream analyses.

### Phylogenetic analysis

Phylogenetic analyses were conducted using HA and NA sequences generated in this study together with representative reference sequences retrieved from GenBank (Supplementarty Tables 1 and 2). Sequences were aligned using MAFFT v.7 [29], and maximum-likelihood phylogenetic trees were inferred using IQ-TREE [30] under the best-fit nucleotide substitution model selected by ModelFinder [31]. Branch support was assessed using 1,000 ultrafast bootstrap replicates. Phylogenetic trees were visualized and edited using FigTree [32].

## Results

### IAV detection and molecular characterization

**Fig. 2:**
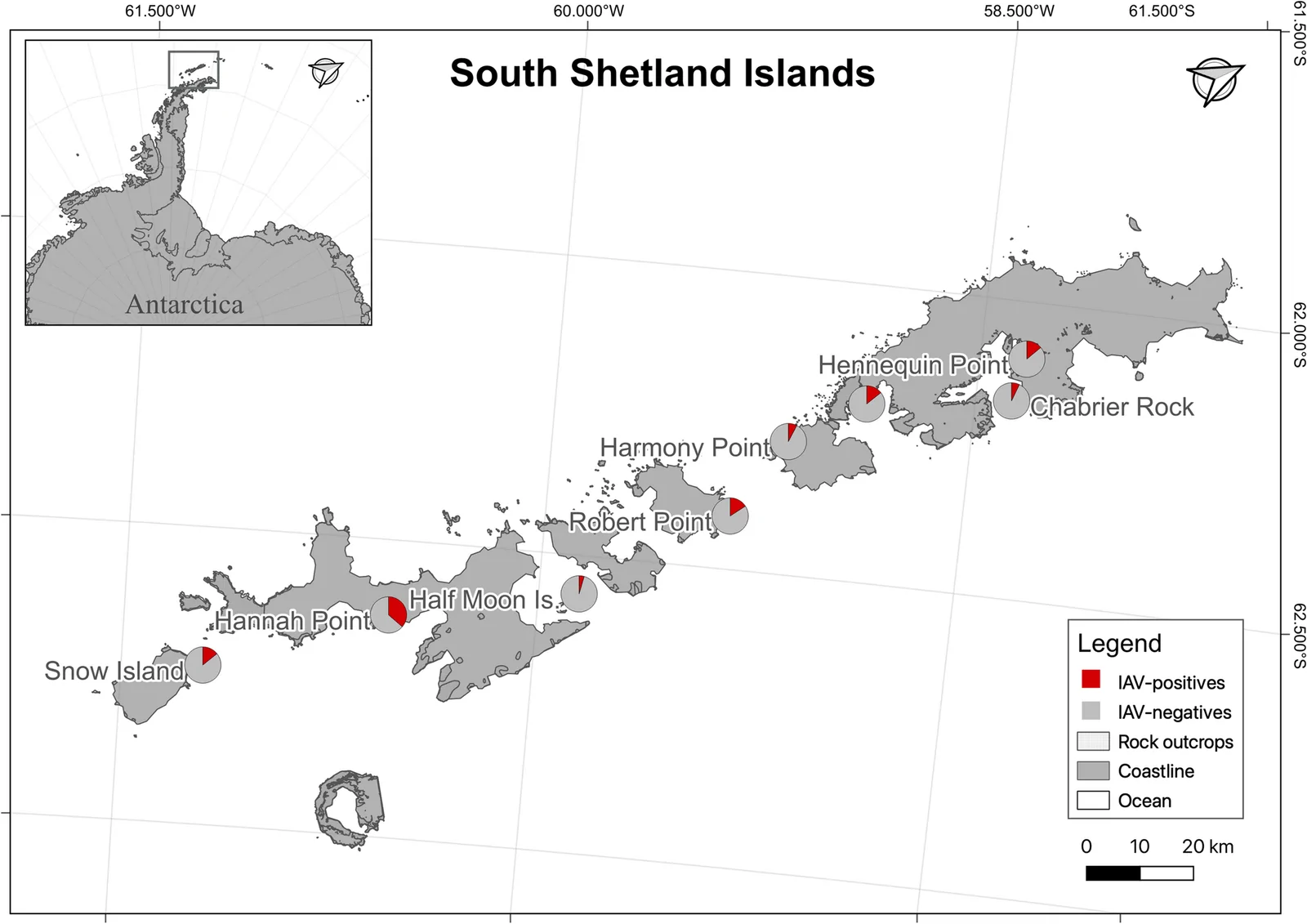
Geographic distribution and prevalence of influenza A virus (IAV) detected during the 2024–2025 austral summer in the South Shetland Islands, Antarctica. Sampling sites are indicated, and pie charts illustrate IAV prevalence (red for positive, grey for negative), with the overall chart size scaled according to the total sample size at each locality. Base map source: SCAR Antarctic Digital Database (ADD v7.11) compiled by the British Antarctic Survey, obtained via the Quantarctica data package (Norwegian Polar Institute) and projected in WGS 84 / Antarctic Polar Stereographic (EPSG: 3031)

Between November and January of the 2024–2025 austral summer, 278 biological samples were collected from Antarctic birds, including 166 cloacal excreta samples, 56 cloacal swabs, and 56 orotracheal swabs. Among the cloacal excreta samples, 25 (~ 15,1%) tested positive for IAV, whereas five 5 cloacal (~ 8,9%) swabs samples were IAV positive (Fig. 2). No orotracheal swab was positive for IAV. Detailed results by sample type, species and collection site are presented in Table 4. Notably, IAV positive samples were identified in all four sampled species (*Pygoscelis papua*, *Pygoscelis antarcticus*, *Stercorarius maccormicki* and *Macronectes giganteus*). Positive samples were distributed across multiple locations in the South Shetland Islands, indicating spatially widespread viral detection within the study area.

**Table 4:** Sampling locations, target bird species, and sample size (n) of samples tested for avian influenza virus (AIV)

Collection site^e^	Species^f^	Sample type	No. samples tested	IAV-positive *n* (%)	H5-positive *n*
Chabrier Rock (King George Is.)	*P. antarcticus*	Excreta	28	2 (7%)	0
	*P. antarcticus*	Swab	22	1 (5%)	1
Half Moon Is.	*P. papua*	Excreta	10	4 (40%)	0
	*S. maccormicki*	Excreta	1	0 (0%)	0
Hannah Pt. (Livingston Is.)	*P. papua*	Swab	13	1 (8%)	0
Harmony Pt. (Nelson Is.)	*P. papua*	Excreta	10	1 (10%)	3
	*P. antarcticus*	Excreta	40	6 (15%)	1
Hennequin Pt. (King George Is.)	*P. papua*	Excreta	33	4 (12%)	2
	*S. maccormicki*	Excreta	30	6 (20%)	0
Robert Pt. (Robert Is.)	*P. papua*	Excreta	14	2 (14%)	0
Two Summit Is.	*M. giganteus*	Swab	21	3 (14%)	1

^e^ *Is*. Island, *Pt*. Point.^f^ P. antarcticus, Pygoscelis antarcticus, *P*. papua, Pygoscelis papua, *S*. maccormicki, Stercorarius maccormicki, *M*. giganteus, Macronectes giganteus

All IAV positive samples were subsequently screened for the H5 hemagglutinin gene using real-time RT-PCR. Of the 30 matrix (M) gene positive samples, eight (~ 26%) were confirmed as H5 positive. Whole-genome sequencing was performed on two H5-positive samples using the Ion Torrent platform. This approach generated complete hemagglutinin (HA) and neuraminidase (NA) gene sequences from one gentoo penguin (*P. papua*) sampled at Hennequin Point, King George Island, and one southern giant petrel (*M. giganteus*) sampled at Two Summit, Nelson Island. Both sequences belong to the HPAIV H5N1, clade 2.3.4.4b. Amino acid analysis of the HA protein revealed a polybasic cleavage site motif (REKRRKR↓GLF) at the HA1–HA2 junction in both isolates.

### Hemagglutinin phylogeny

**Fig. 3:**
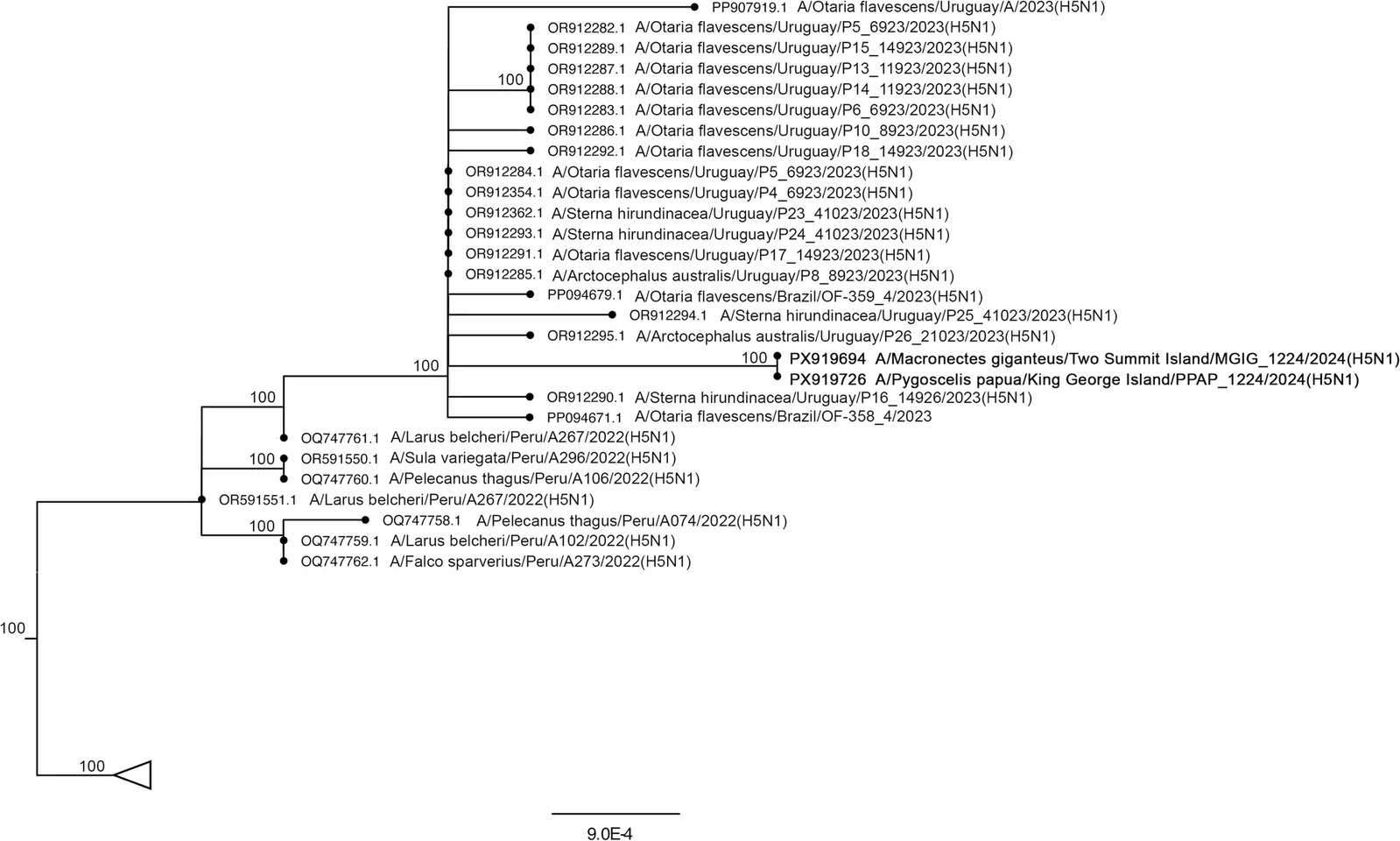
Maximum likelihood phylogenetic tree based on the hemagglutinin (HA) gene of influenza A viruses. Antarctic sequences generated in this study are highlighted and cluster within the highly pathogenic avian influenza virus (HPAIV) H5N1 clade 2.3.4.4b together with South American reference strains. Bootstrap support values ≥ 70% are shown at the nodes, and branch lengths are proportional to the number of nucleotide substitutions per site

Maximum-likelihood phylogenetic analysis of the HA gene showed that both sequences generated in this study clustered within the H5N1 clade 2.3.4.4b lineage (Fig. 3). These sequences are grouped with contemporary South American reference strains, including viruses detected in pinnipeds in Uruguay in 2023 (e.g., OR912295.1), with high bootstrap support (> 90%). The Antarctic sequences formed a well-supported monophyletic cluster within the broader H5N1 clade, whereas several internal nodes showed lower bootstrap support. The HA sequences were deposited in Genbank under the following number accesses: PX919726 (*P. papua*) and PX919694 (*M. giganteus*).

### Neuraminidase phylogeny

**Fig. 4:**
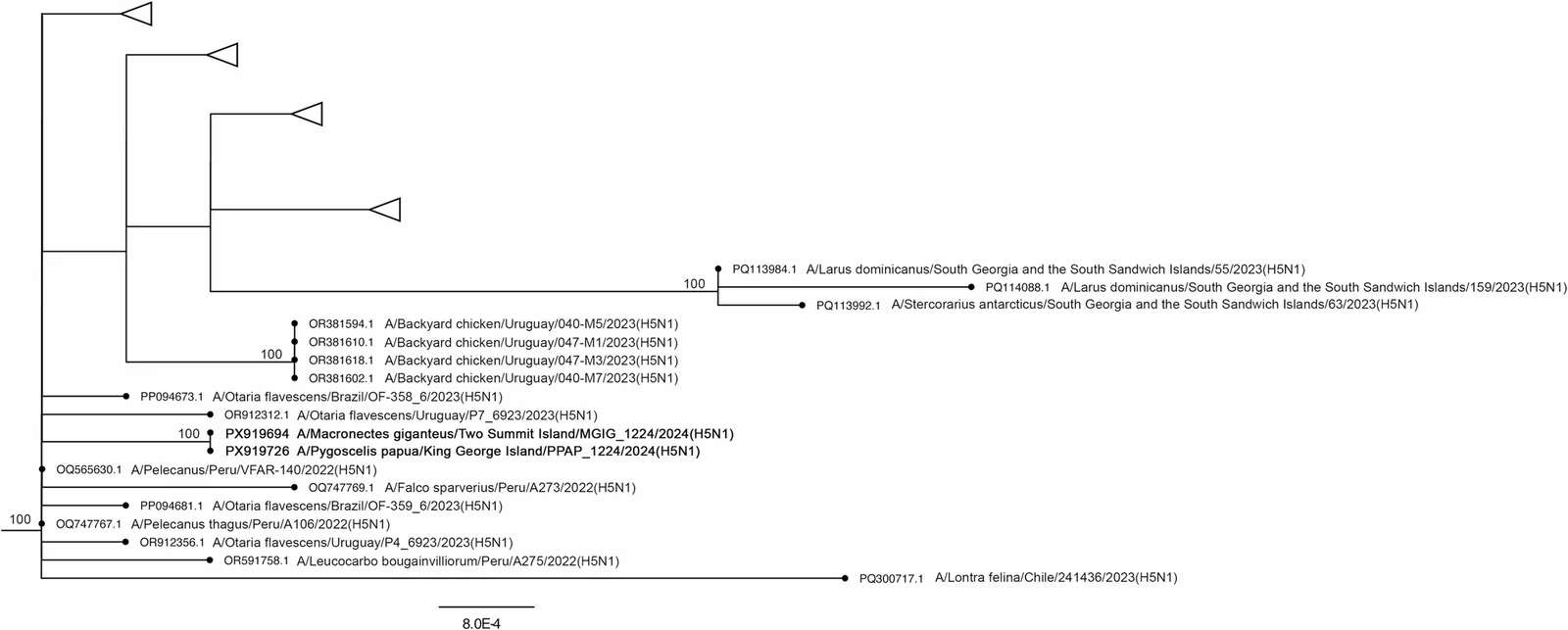
Maximum likelihood phylogenetic tree based on the neuraminidase (NA) gene of influenza A viruses. Antarctic sequences generated in this study are highlighted and cluster within the N1 lineage of the highly pathogenic avian influenza virus (HPAIV) H5N1 clade 2.3.4.4b together with South American reference strains. Bootstrap support values ≥ 70% are shown at the nodes, and branch lengths are proportional to the number of nucleotide substitutions per site

Phylogenetic analysis based on the NA gene sequence showed that the Antarctic sequences clustered within the N1 lineage of HPAIV H5N1 clade 2.3.4.4b (Fig. 4). The isolates are grouped with contemporary South American reference strains with high bootstrap support. The similar phylogenetic placement of the HA and NA segments indicates that both genes share the same evolutionary history, with no evidence of recent reassortment among the Antarctic viruses. The sequences NA were deposited in Genbank under the following number accesses: PX919746 (*P. papua*) and PX919737 (*M. giganteus*).

## Discussion

This study reports the detection and molecular characterization of HPAIV H5N1 in different species of Antarctic seabirds during the 2024–2025 austral summer. The detection of influenza A virus in 30 of 278 samples, of which 8 confirmed as H5-positive, indicates that avian influenza virus circulation in Antarctic avifauna is more widespread than previously documented, even in the absence of conspicuous mortality events at all sampling sites. Noteworthy, the positive samples were obtained either from cloacal swabs or excreta, from all four sampled species collected in different locations in the South Shetland Islands, indicating the widespread of this virus in the region. In addition, our results show that the identified HPAIV belongs to the H5N1 clade 2.3.4.4b lineage.

Whole genomic sequencing showed that the obtained HA sequences contained a polybasic HA0 cleavage site motif (REKRRKR↓GLF), a molecular signature classically associated with highly pathogenic avian influenza viruses [33]. The complete sequencing of the NA gene also supported the classification of these isolates as HPAIV H5N1. Interestingly, these two sequences were obtained from two different species. These findings are consistent with the recent southward expansion of HPAIV H5N1 clade 2.3.4.4b across the Southern Hemisphere. Following its initial detection in South America in October 2022, widespread infection and mortality were reported in wild birds along the Pacific coast, particularly in Chile and Peru [34]. Phylogenetic analyses from these early outbreaks suggested multiple introductions from North America via migratory flyways, establishing a continental source population and raising concern about further dissemination into subantarctic and Antarctic ecosystems [35].

Despite this rapid continental spread, active surveillance conducted in the South Shetland Islands in January 2023 did not detect influenza A virus or H5N1 in seabirds or marine mammals, nor were unusual mortality events observed at that time [36]. Subsequent detections in South Georgia in October–November 2023 and on the Antarctic continent in December 2023, associated with mortality events in seabirds and marine mammals, documented the first confirmed incursions of HPAIV H5N1 into Antarctic ecosystems [13]. Within this temporal framework, the present study provides evidence of continued viral circulation during the 2024–2025 austral summer, supporting a scenario of recent introduction followed by regional dissemination rather than long-term endemic circulation. HPAIV H5N1 was detected in both resident penguin species (*Pygoscelis papua* and *P. antarcticus*) and highly mobile scavenging species (*Stercorarius maccormicki* and *Macronectes giganteus*). South polar skuas undertake long-range and transequatorial migrations, whereas southern giant petrels perform rapid dispersive movements between the Antarctic Peninsula and the southern coasts of South America [9, 37]. These movement patterns support a role for highly mobile species as bridge hosts facilitating viral introduction into Antarctic ecosystems, followed by local amplification within dense seabird breeding colonies.

Phylogenetic analyses further support this interpretation. Antarctic avian strains clustered within the contemporary H5N1 clade 2.3.4.4b lineage currently circulating in the Southern Hemisphere and showed close genetic relatedness to viruses detected in South American birds and marine mammals. In both hemagglutinin and neuraminidase phylogenies, Antarctic isolates grouped with South American strains, reflecting a shared evolutionary origin and temporal continuity of viral circulation. The absence of evidence for recent reassortment in the analyzed genomic segments is consistent with clonal expansion and mirrors genomic patterns reported during recent H5N1 outbreaks in South American marine mammals [38–40]. Additional support for this connectivity comes from the detection of closely related viruses in wide-ranging seabirds sampled in subantarctic regions during 2023, including brown skuas (*Stercorarius antarcticus*) and kelp gulls (*Larus dominicanus*), reinforcing the role of highly mobile seabirds as connectors between South American and Antarctic viral populations [3, 41, 42]. The close phylogenetic relationship between Antarctic isolates and viruses detected in marine-associated hosts further suggests multispecies transmission within interconnected marine ecosystems prior to Antarctic introduction.

The ecological consequences of HPAIV H5N1 circulation in Antarctic ecosystems remain uncertain. Although some species may act as reservoirs with limited clinical impact, others may experience severe disease and high mortality, as observed during outbreaks affecting wild birds and marine mammals in other regions [13]. Antarctic ecosystems, characterized by low species diversity but exceptionally high colony densities, may therefore be particularly vulnerable to the introduction and spread of highly pathogenic avian influenza viruses [43–45]. Taken together, these findings support a framework in which long-distance migratory connectivity facilitates viral introduction into Antarctica, followed by regional dissemination and potential persistence within resident seabird populations. The recovery of complete viral gene sequences from taxa occupying distinct ecological niches demonstrates that highly pathogenic avian influenza viruses can infect a broad range of Antarctic seabirds, underscoring the importance of continued, systematic surveillance to detect viral incursions, assess species-specific susceptibility, and anticipate ecological and evolutionary consequences in a rapidly changing the polar environment.

##  Supplementary material

Below is the link to the electronic supplementary material.

Supplementary Material 1

Supplementary Material 2
